# Transportin HIV-1 by tRNAin and CAr

**DOI:** 10.1186/1742-4690-8-S2-O3

**Published:** 2011-10-03

**Authors:** Lihong Zhou, Elena Sokolskaja, Clare dolly, William James, Sally A Cowley, Ariberto Fassati

**Affiliations:** 1 Wohl Virion Centre, University College London, London, WC1E 4BT, UK; 2 MRC Centre for Medical Molecular Virology, Division of Infection & Immunity, University College London, London, WC1 E4BT, UK; 3 Sir William Dunn School of Pathology, University of Oxford, South Parks Road, Oxford, OX1 3RE, UK

## Background

To replicate, the human immunodeficiency virus type 1 (HIV-1) must access the nucleus of infected cells and integrate into host chromosomes, however little is known about the events occurring post-nuclear entry but before integration. Transportin 3 (Tnp3) is a karyopherin that is required for HIV-1 infection at a step at or post-nuclear entry therefore understanding how Tnp3 works will inform on such early events in the HIV-1 life cycle.

## Materials and methods

To better understand the function of Tnp3 in the HIV-1 life cycle, we have generated Tnp3 knock down cells, including CD4+ T cells and macrophages, and examined the step of the life cycle blocked. We performed pull down assays with viral particles and nuclear export assays using viral and T7 synthesized tRNAs.

## Results

In contrast to current models, we found that Tnp3 promotes HIV-1 integration in different cell types, but has little effect on viral nuclear import. Furthermore Tnp3 bound the viral capsid proteins and tRNAs incorporated into viral particles in pull down assays. The C-terminal region of Tnp3 was required for binding to viral capsid and tRNAs. Interaction between Tnp3, capsid and tRNAs was stronger in the presence of RanGTP, consistent with the possibility thatTnp3 is an export factor for these substrates. In agreement with this interpretation, we found that Tnp3 exports from the nuclei viral tRNAs in a RanGTP-dependent way. Tnp3 also bound and exported from the nuclei some species of tRNAs with a defective 3'CCA end. Depletion of Tnp3 in infected cells resulted in a re-distribution of HIV-1 capsid proteins between nucleus and cytoplasm however HIV-1 bearing the N74D mutation in capsid, which was insensitive to Tnp3 depletion, did not show such a nucleocytoplasmic redistribution of capsid proteins.

## Conclusions

We propose that HIV-1 retains viral tRNAs and some capsid proteins in order to enter the nucleus of infected cells. However such viral components must be displaced to facilitate integration. Tnp3 promotes HIV-1 integration by displacing any capsid and tRNA that remain bound to the viral complex after nuclear entry. The results provide evidence for an unanticipated step of the HIV-1 life cycle that connects the tRNA nucleocytoplasmic trafficking pathway in human cells and viral infection.

